# Challenges and opportunities of deep learning for wearable-based objective sleep assessment

**DOI:** 10.1038/s41746-024-01086-9

**Published:** 2024-04-04

**Authors:** Bing Zhai, Greg J. Elder, Alan Godfrey

**Affiliations:** 1 Department of Computer and Information Sciences, Northumbria University, Newcastle, UK; 2 Northumbria Sleep Research, Department of Psychology, Northumbria University, Newcastle upon Tyne, UK

**Keywords:** Diagnostic markers

## Abstract

In recent years the intersection of wearable technologies and machine learning (ML) based deep learning (DL) approaches have highlighted their potential in sleep research. Yet, a recent study published in NPJ Digital Medicine highlights the generalization limitations of DL models in sleep-wake classification using actigraphy data. Here, this article discusses some of the challenges and opportunities presented by domain adaptation and self-supervised learning (SSL), innovative methodologies that use large-scale unlabeled data to bolster the generalizability of DL models in sleep assessment. These approaches not only improve sleep-wake classification but also hold promise for extending to more comprehensive sleep stage classification, potentially advancing the field of automated sleep assessment through efficient and user-friendly wearable monitoring systems.

## Introduction

Deep learning (DL, Table 1), a subset of machine learning (ML), has significantly impacted the field of automated sleep assessment, especially through the analysis of polysomnography (PSG) data. PSG is the most accurate objective sleep measurement method because it simultaneously assesses multiple physiological parameters, including overnight brain activity, and can classify sleep into distinct stages^1^. DL models trained on clinical PSG data have attained performance levels comparable to human experts, providing clinicians with valuable tools for automated and comprehensive sleep stage analysis^2–5^, across a range of clinical datasets e.g., MESA^6^, SHHS^7^. However, PSG’s suitability for long-term, at-home sleep monitoring is limited due to its intrusive nature. Even headband devices like Dreem™, though less intrusive than traditional PSG technology for brain wave-based sensing, can be cumbersome/uncomfortable during extended wear^8^.

**Table 1:** Terminology and descriptors used in this editorial

Terminology	Description
Actigraphy	Actigraphy uses a non-invasive wearable device to track rest and activity through movement.
Deep learning (DL)	Deep learning is a subset of machine learning (ML) that uses neural networks with many layers to analyze complex patterns in large amounts of data.
Domain adaptation	Domain adaptation is a technique in ML that aims to improve model performance on a target domain by leveraging knowledge from a related but different source domain.
Model overfitting	Model overfitting in ML occurs when a model fits too closely to the training dataset and cannot generalize to new/unseen data
Nearables	A type of smart object that can enhance the interaction with e.g., people and other smart objects. One notable example is a smartphone that can improve the usability and experience of wearing a smart watch.
Photoplethysmography (PPG)	PPG measures capillary blood volume changes by detecting light variations, used for heart rate monitoring.
Polysomnography (PSG)	PSG is the most accurate objective sleep measurement method because it simultaneously assesses multiple physiological parameters, including overnight brain activity, and can classify sleep into distinct stages
Self-supervised learning (SSL)	Self-supervised learning trains models on tasks using the data itself to generate supervisory signals for training on a task without relying on human-provided labels
Signal-to-noise-ratio (SNR)	Signal-to-noise-ratio quantifies the clarity of a signal in a system by comparing its power to that of the background noise, with a higher SNR indicating a clearer signal.

Recent developments in wearable and nearable technologies have made it feasible to monitor sleep in home settings^3,9–11^. Despite advancements, the effectiveness of adopting wearable devices and DL methods for sleep analysis is often hindered by data scarcity, leading to model overfitting^12^. For instance, to estimate sleep parameters, a recent study published in NPJ Digital Medicine by Patterson et al.^13^ evaluated DL models based on actigraphy data in cross-dataset settings and found that those models often struggle with considerable domain discrepancies^13^, which poses challenges for effectively deploying DL models across varied settings and devices. Many wrist-worn devices now feature photoplethysmography (PPG) sensors, alongside actigraphy, indicating their potential for classifying sleep stages^3,4,14^. Nonetheless, many investigations have been conducted on small datasets, yielding limited performance outcomes. Conversely, fields such as natural language processing use abundantly availability datasets to aid the development of sophisticated DL models, such as ChatGPT^15^. That disparity highlights the potential benefits of using large volumes of unlabeled data to enhance sleep monitoring technologies.

## Challenges: Wearable sensing and deep learning

The frequent implementation of DL in various fields is remarkable, yet it encounters two key challenges when applied to sleep assessment through wearable sensing-based methodologies. Namely, (i) small-labelled dataset problem (i.e., data scarcity), and (ii) the balancing act between achieving a high signal-to-noise ratio (SNR, a method that compares the level of a desired signal to the level of background noise) in wearables and maintaining user acceptance for long-term usage.

## Data Scarcity: Annotation and patient availability

In sleep medicine, especially with wearable computing, the development of supervised learning models is impeded by a lack of richly annotated datasets. Obtaining unlabeled data from wrist-worn wearable devices is feasible and pragmatic. However, annotating those data for sleep classification requires simultaneous electroencephalography (EEG) collection and expert medical annotation. That contrasts with fields such as computer vision, where the annotation is more straightforward (i.e., requires less expertise), underscoring the unique difficulties in assembling annotated sleep-based datasets for supervised DL wearable-based algorithms^16,17^.

Furthermore, limited research resources, patient scarcity, and the challenge of recruiting a diverse patient population with varying disease severities exacerbate data imbalances, making models easily overfit to the training dataset, affecting generalizability on unseen populations (i.e., participants were out of the distribution/heterogeneity of the training dataset). That phenomenon is evidenced in the evaluation outcomes presented by Patterson et al., demonstrating that when the training and test datasets originate from the same distribution, the performance of the DL model surpasses that of conventional methods. Assessments based on the proxy signals, such as those from cardiorespiratory signals, reveal distinct patterns in individuals with conditions like sleep apnea^18^, underscoring the need for more diverse data to improve model generalizability.

## Signal to noise ratio: Adequate hardware

The quest for high SNR wearables persists, capable of precisely gauging brain activity with minimal intrusion and optimal comfort^19^. Approaches based on wrist movement and cardiac sensing data may reach a ceiling effect, as peripheral signals might not precisely reflect sleep stages^20^. Traditional scalp and forehead skin-based sensing methods are less perturbed by physiological activities other than the brain^18,21–25^. The advancements made using DL models with PSG data for automated sleep staging analysis highlighted the significant potential of soft textile-based EEG sleep detection devices, such as MUSE™^9,21^. The trade-off between usability and performance remains crucial in developing wearables aimed at sleep stage classification^19^. Moreover, the persistent data scarcity issue remains challenging, necessitating exploration into ML paradigms like self-supervised learning (SSL) and transfer learning as potential avenues to bolster model generalization and adaptability to new tasks.

## Opportunities: Self-supervised machine learning and domain adaptation

In automated sleep analysis, SSL is combined with domain adaptation to become a key strategy for enhancing model generalization^26,27^. Domain adaptation refines models developed in one domain of sleep research (e.g., laboratory sleep patterns) to be applicable in another (e.g., free-living conditions sleep patterns). It overcomes disparities in data volume or quality by discarding irrelevant features and capturing universally recognized patterns, making it a valuable tool for advancing sleep assessment methodologies with limited data. SSL represents a paradigm shift in automated sleep analysis, enabling models to learn from large volumes of unlabelled data through the identification of inherent patterns. This approach is analogous to inferential learning in humans, where understanding is developed through observation rather than explicit instruction (e.g., learning the differences between sleep epochs and similar sleep epochs at different times). By employing *pretext* tasks, such as predicting the next sequence in a series of data points, SSL models can learn general features and patterns relevant to sleep, contributing to the robustness and accuracy of downstream supervised learning tasks classification^28,29^.

The great promise of SSL has been observed across a range of domains in computer vision^30^, natural language processing^31–33^, and speech processing^34^. In automated sleep analysis, with the widespread proliferation of miniature sleep sensing technologies, accumulating substantial quantities of unlabeled data has become increasingly feasible. This development holds the potential to furnish extensive datasets for the training of SSL models, which are frequently structured around an encoder-decoder architecture. The encoders transform raw data into a compact representation, and decoders reconstruct the original data from this representation to learn meaningful patterns without explicit labels. What does that mean? Consider a *pre-train-then-fine-tune* paradigm, the encoder is initially trained to acquire useful representations (features) for downstream sleep-related tasks, such as sleep stage classification and sleep spindle recognition. Subsequently, those learned encoders are frozen, and trained/fine-tuned task-specific classification layers are updated to categorize specific events of interest within a smaller expert-annotated dataset. That approach aims to capture fundamental signal characteristics by learning to discern high-level semantics (e.g., different patterns in sleep data indicate sleep stages, quality, or disturbances) to facilitate effective representation learning. Of further interest is the use of SSL with domain adaptation in integrating those techniques with existing frameworks, potentially enhancing the adaptability and effectiveness of sleep stage classification algorithms across varied data sources and environments.

## Harnessing existing SSL approaches

Various existing framework methodologies like SimCLR^35^, MoCo^36^, SimSiam^37^, and Barlow Twins^38^, offer universally adaptable frameworks that could seamlessly extend into sleep monitoring, warranting investigations into their efficacy. For instance, a recent study using accelerometer data alone from over 96,000 UK Biobank participants has shown the effectiveness of SSL for three-stage sleep classification and achieved an F1 score of 0.573 ± 0.12, representing a 7.1% improvement over the baseline model that did not incorporate SSL pre-training, as validated through internal evaluations^39^. This outcome challenges previous assumptions regarding the feasibility of sleep stage classification using accelerometer data only. That method, crucial in a domain with limited labelled data, emphasizes the effectiveness of general representations learned through SSL for sleep stage classification.

In conclusion, the study by Patterson et al. highlighted the vulnerability of basic DL models to overfitting, particularly when applied to specific datasets, data preprocessing methodologies, and PSG annotation styles as demonstrated through a single cross-dataset evaluation. The effort to accumulate large-scale datasets of sleep stages, annotated by experts from raw data gathered through wearable devices continues to present a significant challenge. Nonetheless, DL has shown considerable promise in a single dataset setting. Hence, using vast amounts of unlabeled raw data from wearables and exploring sophisticated model architectures to improve generalizability, like integrating SSL and domain adaptation, offers a promising path for advancing long-term sleep assessment.
